# Risk of Selected Fetal Adverse Pregnancy Outcomes at Advanced Maternal Age: A Retrospective Cohort Study in Debre Markos Referral Hospital, Northwest Ethiopia

**DOI:** 10.1155/2020/1875683

**Published:** 2020-12-24

**Authors:** Bikila Tefera Debelo, Melaku Hunie Asratie, Abayneh Aklilu Solomon

**Affiliations:** ^1^Department of Midwifery, College of Medicine and Health Science, Ambo University, Ambo, Ethiopia; ^2^Department of Women's and Family Health, School of Midwifery, College of Medicine and Health Sciences, University of Gondar, Gondar, Ethiopia; ^3^Department of Clinical Midwifery, School of Midwifery, College of Medicine and Health Sciences, University of Gondar, Gondar, Ethiopia

## Abstract

**Introduction:**

Pregnancy at an advanced maternal age is defined as pregnancy at 35 years or older. Today, women postpone pregnancy due to different socioeconomic and personal reasons. However, there was limited evidence on fetal adverse outcomes' association with pregnancy at an advanced maternal age in Ethiopia and particularly in the study area. This study was aimed at assessing the effect of pregnancy at an advanced age on selected neonatal adverse pregnancy outcomes in Debre Markos Referral Hospital, Ethiopia, 2019.

**Methods:**

Institution-based retrospective cohort study was conducted on 303 exposed (35 years and older) and 604 nonexposed (20–34 years old) immediate postpartum women who delivered at Debre Markos Referral Hospital after 28 weeks of gestation. All exposed women who fulfilled the inclusion criteria were sampled, and systematic random sampling was employed for those in the nonexposed group. The data were collected from 1st of July to 30th of December, 2019, by face-to-face interview and extraction from maternal chart using a structured questionnaire and data extraction checklist, respectively. Binary logistic regression (bivariate and multivariable) model was fitted, and wealth index was analyzed by principal component analysis. Adjusted relative risk with respect to 95% confidence interval was employed for the strength and directions of association between advanced maternal age and selected adverse pregnancy outcomes, respectively. *P*-value of <0.05 was used to declare statistical significance.

**Results:**

The incidence of adverse neonatal outcomes including stillbirth, preterm birth, and low birth weight in the advanced maternal age group was 13.2%, 19.8%, and 16.5%, respectively. The incidence of stillbirth, preterm birth, and low birth weight in the nonexposed group was 3.1%, 8.4%, and 12.4%, respectively. The advanced maternal age group had three times the risk of stillbirth compared with the nonexposed group (ARR = 3.14 95% CI (1.30–7.00)). The advanced maternal age group had 2.66 times the risk of delivering preterm fetus (ARR = 2.66 95% CI (1.81–3.77)) compared with the younger counterparts. Low birth weight was not significantly associated with pregnancy at an advanced maternal age.

**Conclusion:**

Fetal adverse outcomes including stillbirth and preterm birth were significantly associated with pregnancy at an advanced maternal age.

## 1. Background

Advanced maternal age (AMA) is defined as pregnancy at a maternal age of 35 years or older [1]. AMA pregnancy varies across countries, from 33.4% in Norway to 11.4% in Taiwan [2, 3]. The proportion of pregnancy at AMA in three different continents, Latin America, Middle East Asia, and Africa, is 12.3% [2].

Different reasons for postponing pregnancy were described by different studies. Occupational and socioeconomic, personal and marriage problems, childbearing desire and conception issues, and infertility were among the reasons for late-age pregnancy [4].

Pregnancy at AMA is associated with different adverse neonatal outcomes; however, there are contradicting ideas about those adverse outcomes. Pregnancy at AMA is associated with stillbirth, even though the mechanism is not entirely clear [5].

Pregnancy in women of advanced maternal age is complicated by intrauterine fetal death (IUFD) and neonatal death [6]. Perinatal adverse outcomes like preterm birth, early neonatal death, low birth weight, neonatal intensive care unit (NICU) admission, and APGAR score of less than seven at five minutes are significantly associated with AMA [2].

The adverse outcome in AMA pregnancy stems from inadequate cardiovascular adaptation during pregnancy, which impedes the hemodynamic changes for supporting the fetus [7]. This might explain the fact that pregnancy in AMA is associated with intrauterine growth restriction and placental abruption [8]. Pregnancy-associated complications in AMA affect the future glucose metabolism of the newborn [9].

Biologically, according to the royal college of obstetricians and gynecologists, the optimum period for childbearing is between 20 and 35 years of age [10]. Pregnancy at later maternal age is an emerging public health issue. Women should be supported, rather than constrained, in their life choices. However, both women and society need to be aware of the possible problems that older mothers may encounter. There is an urgent need for better public information on the issues surrounding later maternity. Despite all these, the effect of AMA pregnancy on pregnancy outcomes is contradictory and reported differently by different studies [11].

Ethiopia has done a tremendous job in preventing teenage pregnancy; however, the pregnancy in older-aged women is given less concern even though postponing pregnancy due to the educational and socioeconomic condition is increasing. Pregnancy in AMA is studied and approached poorly in Ethiopia. A significant number of mothers included in the Ethiopian Demographic Health Survey (EDHS) were AMA although their birth outcomes were not assessed. Therefore, this study aims at assessing the effect of advanced maternal age pregnancy on neonatal outcomes.

## 2. Methods

### 2.1. Study Design, Setting, and Period

An institution-based retrospective cohort study was conducted in Debre Markos Referral Hospital, Northwest Ethiopia, from 1st July to 20th December 2019. Debre Markos is a city located 300 kilometers far from Addis Ababa, the capital of Ethiopia, and 256 km from Bahir-Dar, the capital of Amhara National Regional State. The hospital provides health services to more than 3.5 million populations. Currently, about 100 health centers and two district hospitals are available in the catchment area of the referral hospital. There are 109 nurses, 3 health officers, 16 general practitioners, and 2 emergency surgeons and 28 specialists. The gynecologic and obstetric ward has 36 midwives, 14 general practitioners, and 7 gynecologists. The ward has a total of 60 beds. According to the 2011 E.C. six-month report of Debre Markos referral hospital, 3005 women had delivered at the hospital of which 5% were stillbirth.

### 2.2. Source and Study Population

Women aged 20–34 years and 35 years or older at Debre Markos Referral Hospital catchment area were the source population for the exposed and nonexposed groups, respectively. All women aged between 20 and 34 years and 35 years or older who gave birth after 28 weeks of gestation at Debre Markos Referral Hospital during the data collection were the study population for the exposed and the nonexposed groups, respectively. All postpartum women aged 20 years or older who gave multiple births and women with medical complications, including chronic hypertension, diabetes mellitus, CHF, and thyrotoxicosis, were excluded from the study.

### 2.3. Sample Size Determination and Sampling Procedures

The sample size was calculated by using epi info software version 7.2.1.0. The assumptions for the calculation were 95% confidence level, 80% power, ratio of the nonexposed to the exposed group of 2, and 10% nonrespondent rate. A total of 912 mothers were included in the study by adding 10 % nonrespondent rate, 304 from the advanced maternal age group and 608 women from mothers aged 20 to 34 years [1, 12].

Systematic random sampling was employed for the nonexposed group, and all advanced maternal age mothers who fulfilled the inclusion criteria were included in the exposed group. Two to one ratio of nonexposed and exposed groups was used to increase the study power making the sample size of nonexposed group twice the exposed group.

All consecutive women aged 35 and above who fulfilled the inclusion criteria were selected as an exposed group. As for the nonexposed group, 20–34 aged mothers were selected by systematic random sampling, *K* was calculated by dividing the six-month report of Debre Markos Referral Hospital delivery service, which was 3005 deliveries by the total sample for the nonexposed group; that is, *K*=(3005/608)=5. Therefore, a random sample was taken among the first five women and every five women were included in the nonexposed group. For every exposure, two nonexposures were included by a systematic random sampling technique.

### 2.4. Study Variables and Measurement

  Dependent/outcome variables are as follows: fetal outcomes: preterm birth, low birth weight, and stillbirth.
  Exposure variable is maternal age. Other predictor variables are as follows: gravidity, parity, alive children, educational level, residence, marital status, occupation, sex of the infant, number of prenatal visits, previous pregnancy adverse outcomes, health insurance, and wealth quintile.
  Advanced maternal age pregnancy is defined as pregnancy in women aged 35 years or older [2].
  Exposed group includes women at an advanced age [1].
  Nonexposed group includes women aged 20–34 years [1].
  Preterm birth is any birth before 37 completed weeks of gestation or at greater than 28 weeks [13].
  Stillbirth is a fetus with no signs of life before the complete expulsion or extraction from its mother and after a predefined duration of gestation; after delivery, it is confirmed that the fetus does not show any evidence of life and cannot be resuscitated [14].
  Low birth weight is a birth weight of less than 2500 grams regardless of the gestational age [15].

### 2.5. Data Collection Instruments and Quality Assurance Measures

Structured questionnaires and data extraction checklists developed based on literature with modification to this study setting were used as a data collection tool. The questionnaires were developed in English and those collected from the mother directly were translated to the local language, Amharic, by language expertise and back to English for consistency.

Exposure variable which was maternal age and other sociodemographic and economic factors were collected from mothers by face-to-face interview, whereas neonatal outcomes were extracted from the mothers' chart. Data was collected by five final year MSc students, three males and two females. They are all instructors at local universities. The data was collected during the postpartum period and at the time of discharge daily. Training of data collectors and a supervisor was made to ensure the quality of the collected data. Principal investigator and supervisors had made spot-checking and reviewed all the completed questionnaires and checklists to ensure completeness and consistency of the collected information. The data collection process was supervised by two on-site supervisors, and data entry was carried out by the principal investigator.

### 2.6. Data Processing and Analysis

The data gathered and extracted through the structured questionnaire and checklists, respectively, were entered to EPI- DATA version 4.6.0.0, coded, cleaned, and exported to SPSS version 23 for analysis.

Descriptive statistics comparing the neonatal outcomes across the two groups were presented by frequency and percentage. The wealth quintile was analyzed by using principal component analysis (PCA) for urban and rural participants separately and the final outputs were grouped based on the EDHS 2016 wealth quintile grouping.

The women were classified into maternal age categories: 20–34 years old (nonexposed group) and 35 years old or older, and each pregnancy outcome was dichotomized as “yes or no” for analysis. The following potential confounders were included in the adjusted models: sociodemographic, economic, and obstetric factors. Only variables that reached a *P*-value of less than 0.2 in the bivariable logistic regression analysis were included in the multivariable logistic regression model. A *P*-value less than 0.2 and 0.05 was taken as a cut of value to be significant in bivariable and multivariable logistic regressions, respectively. Associations between maternal age and neonatal adverse outcomes were assessed, and its strength is presented using adjusted relative risk (aRR) and 95% confidence intervals.

Both bivariable and multivariable logistic regressions were used to assess the association between selected adverse neonatal pregnancy outcomes and exposure variable, maternal age.

Both crude and adjusted relative risks were calculated from the crude and adjusted odds ratio, respectively, which in turn were obtained from the logistic regression outputs. The formula used to calculate the relative risk was RR=OR/(1 − *I*_*o*_)+(*I*_*o*_ × OR) [16, 17], where *I*_*o*_ is incidence in the nonexposed group, and OR represents the crude and adjusted depending on the relative risk calculated (crude OR for crude RR and adjusted OR for adjusted RR). Similarly, 95% confidence interval for the RR was obtained by applying the same correction to the confidence interval bounds of the OR.

## 3. Results

### 3.1. Socioeconomic and Demographic Characteristics of the Study Participants

In this study, a total of 912 women were included, 608 from the nonexposed group and 304 from the exposed group. The response rate for the nonexposed group was 99.2%, where four sampled women declined to participate, and 99.7% for the exposed group, where one woman declined to participate.

About 73% of the nonexposed group was of urban residence, and 60.7% of the exposed group was of rural residence. More than one quarter (29.5%) of the nonexposed group had a college and/or above education, and one in six of them was a government employee. More than three fourth (76.9%) of the exposed group had no formal education, and more than half (53.1%) of them were housewives (Table 1).

**Figure 1:**
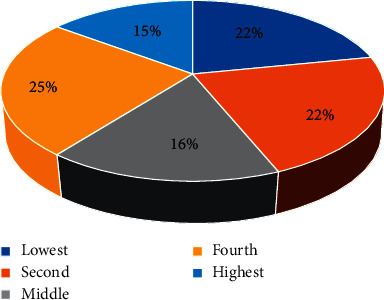
Distribution of wealth quintile of the participants in Debre Markos referral hospital, Northwest Ethiopia, 2019 (*N*_E_ = 303 and *N*_Ne_ = 604).

More than one fifth (nearly 22%) of the respondents' wealth quintile falls in the lowest category of the 2016 Ethiopian Demographic Halth Survey's wealth quintile classification, and about 25% of the participants' wealth quintile was fourth (Figure 1).

### 3.2. Obstetric Characteristics of the Study Participants

More than half of the nonexposed group were impregnated with their first child, and 92.4% of the exposed group had become pregnant more than once. More than one third (34.7%) of the exposed group were grand multiparous. About 93.4% of the exposed group did have ANC follow-up, and 97% of the nonexposed group had ANC follow-up (Table 2).

### 3.3. Fetal Pregnancy Outcomes

**Figure 2:**
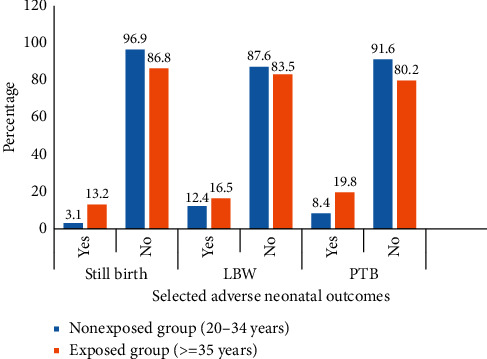
Distribution of selected neonatal adverse pregnancy outcomes over grouped maternal age in Debre Markos referral hospital, Northwest Ethiopia, 2019 (*N*_E_ = 303 and *N*_Ne_ = 604).

The incidence of stillbirth among the AMA and nonexposed groups was 13.2% and 3.1%, respectively. The incidence of PTB and LBW in the AMA group was 19.8 and 16.5%, respectively (Figure 2).

### 3.4. Selected Adverse Neonatal Pregnancy Outcomes and Pregnancy at an Advanced Maternal Age

Binary logistic regression was fitted to assess the association between maternal age and selected adverse neonatal pregnancy outcomes and other factors. All factors with *P*-value ≤ 0.2 were taken to multivariate analysis along with maternal age for further analysis, thus controlling for potential confounding effects. A backward LR method of analysis was applied and model fitness was checked by Hosmer-Lemeshow goodness-of-fit.

Maternal age was associated with stillbirth in bivariable logistic analysis; other factors including place of residence, parity, gravidity, mother and husband's educational status, health insurance, wealth quintile, ANC follow-up, and mother and husband's occupational status were also associated with stillbirth and hence included in multivariable analysis. The AMA group had three times the risk of stillbirth compared with the nonexposed group (ARR = 3.14 95% CI (1.30–7.00)) (Table 3).

Low birth weight was not significantly associated with maternal age on binary logistic level (Table 3).

Advanced maternal age pregnancy was significantly associated with PTB and so were other socioeconomic and obstetrics factors like number of alive children, wealth index, husband's occupational status, gravidity, husband's educational status, mother's educational status, parity, ANC follow-up, previous adverse pregnancy outcomes, health insurance, and place of residence at binary logistic analysis level. Maternal age was entered into the multivariable logistic analysis alongside with those factors associated with preterm birth to control for confounding effects. The AMA group had 2.66 times the risk of delivering preterm fetus (ARR = 2.66 95% CI (1.81–3.77)) compared with the younger counterparts (Table 3).

## 4. Discussion

This study was aimed at assessing the fetal adverse outcomes associated with advanced maternal age. Accordingly, the risks of stillbirth and low birth weight were higher in pregnancy at an advanced maternal age compared with the younger counterparts. Low birth weight, however, was not significantly associated with pregnancy at an advanced maternal age.

The incidence of stillbirth in the exposed group is higher compared with the nonexposed group, 13.2% versus 3.1%. This incidence of stillbirth in the exposed group is lower compared with the study done in Ghana, which reported incidence of still birth in the exposed group to be 20.7% and the in nonexposed group to be 5% [1]. The difference in incidence could be due to the difference in study design and study setting implicating different maternal and fetal and/or neonatal care practices. This difference in maternal healthcare practice across the two countries could contribute to the difference in the incidence of still birth.

This study also reported the incidence of PTB to be 19.8% in the exposed group and 8.4% in the nonexposed group. This finding is lower than the 77.6% of PTB incidence in the AMA group reported by the prospective cohort study done in Ghana [1]. This variation could be due to the difference in the study design and setting.

The current study has also reported the incidence of LBW in the exposed group to be 16.5%. This incidence is lower than the incidence of LBW reported by the study done in Ghana, which was 44.8% [1]. This variation could be explained by the difference in the study setting.

The current study found that the AMA group had three times the risk of stillbirth compared with the nonexposed group. This finding is in accord with the study done in Denmark, Risk of Adverse Pregnancy Outcomes at Advanced Maternal Age [18], a multicountry assessment of AMA and pregnancy outcomes [2]. Similarly, the current finding is consistent with a prospective study done in Ghana which concluded the AMA increased the risk of stillbirth [1]. The decreased placental perfusion in mothers with increased age could explain this increased risk of stillbirth in the AMA group [19].

Different studies, however, had different conclusions about the association between AMA and stillbirth. Among the studies that are in contrast with the current study are the studies done in Oman which stated that there is no increased risk of stillbirth in AMA group compared with nonexposed group [20], a study done in three UK hospitals [21]. The study done on the risk of adverse pregnancy outcomes at advanced maternal age had also concluded that no statistically significant difference was observed for pregnant women aged ≥40 years [18].

According to the current study, low birth weight is not significantly associated with maternal age. This finding is consistent with studies done in high-income developing countries which stated that there is no significant difference in low birth weight between AMA and nonexposed group [22]. The studies done in Yaoundé, Cameroon [12], and prospective cohort study in Ghana [1], however, concluded that neonates from women of the AMA group are at increased risk of low birth weight. This difference can be explained by the difference in study setting pertaining to variation in clinical advancement and obstetrical practice.

This study has also revealed that preterm delivery has increased in the AMA group compared with the younger aged women. The AMA group had 2.66 times the risk of delivering preterm fetus compared with the younger counterparts. This finding supports the prospective cohort study done in Ghana which concluded that preterm birth was one of the other independent factors associated with the AMA group [1]. This increased risk of PTB could be attributed by the increased hypoxic placenta originating from placental insufficiency and incomplete maternal artery remodeling, both of which can cause PTB either iatrogenically or spontaneously [23].

## 5. Limitations of the Study

This is a baseline study and is also the first to be conducted in the country; hence, future studies on longitudinal cohorts including all adverse fetal outcomes would help gaining additional insights into pregnancy at an advanced maternal age and adverse fetal pregnancy-related outcomes.

## 6. Conclusion

According to the current study, the incidence of adverse fetal pregnancy outcomes was higher in the AMA group compared with the nonexposed group. The fetal adverse outcomes were statistically associated with advanced maternal age. AMA pregnancy also increases the risk of fetal adverse outcomes (stillbirth and preterm birth) compared with the younger counterparts, and on the contrary, LBW was not significantly associated with AMA.

## Figures and Tables

**Table 1:** Distribution of sociodemographic characteristics over grouped maternal age in Debre Markos referral hospital, Northwest Ethiopia, 2019 (*N*_E_ = 303 and *N*_Ne_ = 604).

Characteristics	Nonexposed group (%)	Exposed group (%)
*Place of residence*		
Urban	443 (73.3)	119 (39.3)
Rural	161 (26.66)	184 (60.7)

*Maternal educational status*		
No formal education	164 (27.2)	233 (76.9)
Primary education	108 (17.9)	25 (8.3)
Secondary education	154 (25.5)	22 (7.3)
College and above	178 (29.5)	23 (7.6)

*Maternal occupational status*		
Government employee	118 (19.5)	20 (6.6)
Self-employed	157 (26)	43 (14.2)
Housewife	288 (47.7)	161 (53.1)
Others^1a^	41 (6.8)	79 (26.1)

*Marital status*		
Married	585 (96.9)	293 (96.7)
Others^1b^	19 (3.1)	10 (3.3)

*Educational status of the husband*		
No formal education	145 (24.8)	182 (62.1)
Primary education	105 (18)	46 (15.7)
Secondary education	149 (25.5)	30 (10.2)
College and above	186 (31.8)	35 (12)

*Occupational status of the husband*		
Government employee	150 (25.6)	45 (15.4)
Farmer	165 (28.2)	184 (62.8)
Others^1c^	(270) 44.7	64 (21.8)

*Health insurance*		
Yes	169 (28)	172 (56.8)
No	435 (72)	131 (43.2)

*N*_E_-total in exposed group, *N*_Ne_-total in nonexposed group. ^1a^farmer, un-employed, student ^1b^single, divorced, widowed, separated ^1c^self-employed, un-employed, student.

**Table 2:** Distribution of obstetrics related characteristics over grouped maternal age in Debre Markos referral hospital, Northwest Ethiopia, 2019 (*N*_E_ = 303 and *N*_Ne_ = 604).

Characteristics	Nonexposed group (%)	Exposed group (%)
*Gravidity*		
Primigravida	318 (52.6)	23 (7.6)
Multigravida	286 (47.4)	280 (92.4)

*Preceding birth interval (N*_*E*_ *=* *277 and N*_*Ne*_ *=* *263)*		
<2 years	29 (11)	20 (7.2)
≥2 years	234 (89)	257 (92.8)

*Parity*		
Primiparous	341 (56.5)	26 (8.6)
Multiparous	249 (41.2)	172 (56.8)
Grand multiparous	14 (2.3)	105 (34.7)

*ANC follow-up*		
Yes	586 (97)	283 (93.4)
No	18 (3)	20 (6.6)

*Number of ANC Visit (N*_*E*_ *=* *283 and N*_*Ne*_ *=* *586)*		
Less than 4	188 (32.1)	111 (39.2)
4 and more	398 (67.9)	172 (60.8)

*Iron folate supplemented (N*_*E*_ *=* *283 and N*_*Ne*_ *=* *586)*		
Yes	562 (96)	274 (96.8)
No	24 (4)	9 (3.2)

*Previous adverse px outcomes*		
Yes	113 (18.7)	150 (49.5)
No	491 (81.3)	153 (50.5)

**Table 3:** Bivariate and multivariable analysis of maternal age and other variables associated with selected fetal adverse outcomes in Debre Markos referral hospital, Northwest Ethiopia, 2019 (*N*_E_ = 303 and *N*_Ne_ = 604).

	Stillbirth^3a^		Crude RR (95% C.I.)	Adjusted RR (95% C.I.)
	No	Yes		
*Maternal age*				
Nonexposed group	585	19	1	1
AMA group	263	40	4.22 (2.53–6.75)^*∗∗*^	3.14 (1.30–7.00)^*∗*^
	LBW			
	No	Yes		
Nonexposed group	529	75	1	
AMA group	253	50	1.33 (0.96–1.83)	
	PTB^3b^			
	No	Yes		
Nonexposed group	553	51	1	1
AMA group	243	60	2.36 (1.68–3.23)^*∗∗*^	2.66 (1.81–3.77)^*∗∗*^

^3a^Adjusted for: Place of residence, maternal occupational status, maternal educational status, gravidity, parity, ANC follow-up, previous adverse pregnancy outcome(s), wealth quintile, health insurance. Hosmer-Lemeshow Goodness-of-fit *P*-value = 0.252. ^3b^Adjusted for: number of alive child(ren), wealth quintile, husband occupational status, gravidity, husband educational status, maternal educational status, parity, ANC follow-up, previous adverse pregnancy outcomes, health insurance, place of residence. CI, Confidence Interval; RR, Risk Ratio. Hosmer-Lemeshow Goodness-of-fit, *P*-value = 0.974. ^*∗*^*P*-value <0.05, ^*∗∗*^*P*-value <0.001 RR=OR/(1 − *I*_*o*_)+(*I*_*o*_ × OR), *I*_*o*_ incidence in the nonexposed group, OR- Odds ratio.

## Data Availability

The datasets used and/or analyzed during the current study are available from the corresponding author on reasonable request.

## Abbreviations

AMA: Advanced maternal age
ARR: Adjusted relative risk
PTB: Preterm birth
SB: Stillbirth.
